# Generative AI-based knowledge graphs for the illustration and development of mHealth self-management content

**DOI:** 10.3389/fdgth.2024.1466211

**Published:** 2024-10-07

**Authors:** Marc Blanchard, Vincenzo Venerito, Pedro Ming Azevedo, Thomas Hügle

**Affiliations:** ^1^ Department of Rheumatology, University Hospital (CHUV) and University of Lausanne, Lausanne, Switzerland; ^2^ Rheumatology Unit, Department of Precision and Regenerative Medicine and Ionian Area (DiMePre-J), University of Bari Aldo Moro, Bari, Italy

**Keywords:** fibromyalgia, knowledge graph, chronic musculoskeletal pain syndromes, artificial intelligence, large language model, ChatGPT, mHealth (mobile health)

## Abstract

**Background:**

Digital therapeutics (DTx) in the form of mobile health (mHealth) self-management programs have demonstrated effectiveness in reducing disease activity across various diseases, including fibromyalgia and arthritis. However, the content of online self-management programs varies widely, making them difficult to compare.

**Aim:**

This study aims to employ generative artificial intelligence (AI)-based knowledge graphs and network analysis to categorize and structure mHealth content at the example of a fibromyalgia self-management program.

**Methods:**

A multimodal mHealth online self-management program targeting fibromyalgia and post-viral fibromyalgia-like syndromes was developed. In addition to general content, the program was customized to address specific features and digital personas identified through hierarchical agglomerative clustering applied to a cohort of 202 patients with chronic musculoskeletal pain syndromes undergoing multimodal assessment. Text files consisting of 22,150 words divided into 24 modules were used as the input data. Two generative AI web applications, ChatGPT-4 (OpenAI) and Infranodus (Nodus Labs), were used to create knowledge graphs and perform text network analysis, including 3D visualization. A sentiment analysis of 129 patient feedback entries was performed.

**Results:**

The ChatGPT-generated knowledge graph model provided a simple visual overview with five primary edges: “Mental health challenges”, “Stress and its impact”, “Immune system function”, “Long COVID and fibromyalgia” and “Pain management and therapeutic approaches”. The 3D visualization provided a more complex knowledge graph, with the term “pain” appearing as the central edge, closely connecting with “sleep”, “body”, and “stress”. Topical cluster analysis identified categories such as “chronic pain management”, “sleep hygiene”, “immune system function”, “cognitive therapy”, “healthy eating”, “emotional development”, “fibromyalgia causes”, and “deep relaxation”. Gap analysis highlighted missing links, such as between “negative behavior” and “systemic inflammation”. Retro-engineering of the self-management program showed significant conceptual similarities between the knowledge graph and the original text analysis. Sentiment analysis of free text patient comments revealed that most relevant topics were addressed by the online program, with the exception of social contacts.

**Conclusion:**

Generative AI tools for text network analysis can effectively structure and illustrate DTx content. Knowledge graphs are valuable for increasing the transparency of self-management programs, developing new conceptual frameworks, and incorporating feedback loops.

## Introduction

Online self-management programs, also refered as Digital Therapeutics (DTx), are increasingly recognized and endorsed in clinical guidelines (1). These applications, have been proven effective in chronic pain syndromes such as fibromyalgia, e.g., by applying Cognitive Behaviour Therapy (CBT) or physical exercise instructions (2). In a recent landmark study, digital acceptance and commitment therapy significantly improved clinical outcomes in fibromyalgia patients (3). In some countries, DTx are reimbursed by health insurers, such as Digital Health Applications (Digitale Gesundheits Anwendungen, DIGAs) in Germany. Online self-management programs with proven efficacy are also available for other indications, including immune-mediated diseases such as rheumatoid arthritis or chronic bowel inflammation (4). However, they often vary in duration, intensity, and content, making comparisons challenging (5). A more detailed presentation of their content would facilitate better assessment of their impact and enable necessary improvements.

To develop personalized treatment strategies, it's essential to integrate fragmented knowledge across biological scales, from molecular factors to clinimetrics and phenotypic outcomes. However, relevant data for health care self-management is dispersed across various domains. Evidence can be found in publications or electronic health records, whereas data on usability, adherence and popularity is may be better reflected in social media. Knowledge graphs, can mitigate this fragmentation by structurally integrating diverse data sources into a cohesive framework, thereby facilitating precision medicine in complex diseases (6).

The advent of Generative Artificial Intelligence (AI), including large language models like ChatGPT4, offers new avenues for semantic text analysis. Through knowledge graphs and text network analysis, these AI tools can elucidate concepts within texts and map complex relationships e.g., in the field of drug discovery (7). This capability can be leveraged to structure knowledge representation, aiding in hypothesis generation, data exploration, and discovery of new insights, thus enhancing scientific research and analysis.

In this proof-of-concept study, we explore the application of knowledge graphs based on large language models (LLM) in a mobile health (mHealth) intervention. We use an online program that we have created for patients with fibromyalgia or post-viral fibromyalgia-like symptoms as the basis for the content (8). We hypothesize that visualizing app content in an interconnected graph-based format will increase transparency and enable users to navigate and understand the information more intuitively. This approach could not only improve health outcomes and app experiences but also facilitate comparisons, such as in clinical study results.

## Methods

### Data source

For the basic structure of this online self-management program and the user interface, we conducted an online survey among patients diagnosed with long COVID. Focus groups were performed to discuss content and engagement with a rehabilitation program provided by native mobile app (8). The mHealth program was tested and further developed as an adjuvant intervention in patients undergoing a multimodal inpatient program for chronic musculoskeletal pain syndromes, including fibromyalgia or fibromyalgia-like syndromes and long Covid. For this we created five digital personas through clustering by unsupervised machine learing as reported elsewhere (9). In brief, our cohort consisted of 201 patients with chronic musculoskeletal pain (10). 78% of the patients fullfilled the ACR2010 criteria for fibromyalgia or the Fibromyalgia Rapid Screening Tool (FiRST), 58% suffered from depression and 22% were diagnosed with a concurrent chronic inflammatory rheumatic disease (IMRD). A substantial number of those patients reported a deterioration of their symptoms after a SARS-Cov2 infection or vaccination, respectively. Their evaluations included a wide range of questionnaires such as the Brief Pain Index (BPI) for pain intensity and impact, the Oswestry Disability Index (ODI) for function, the Tampa Scale for Kinesiophobia (TSK), the Pain Catastrophizing Scale (PCS), and the Fear-Avoidance Beliefs Questionnaire for physical activity and work (FABQ-P and FABQ-W). We also assessed specific behavioral profiles associated with pain using the Patterns Of Activity Measure, depression and anxiety via the Hospital Depression and Anxiety Scales (HDS and HAS). Additionally, the Toronto Alexithymia Scale-20 (TAS-20) and the Pain Disability Questionnaire (PDI) were administered. Sleep quality was assessed by actigraphy.

All procedures involving human participants were in accordance with ethical standards, and written consent was obtained from all individual participants included in the study. Ethical approval for this research was granted by the relevant committee.

### Mhealth self-management program

**Figure 1:**
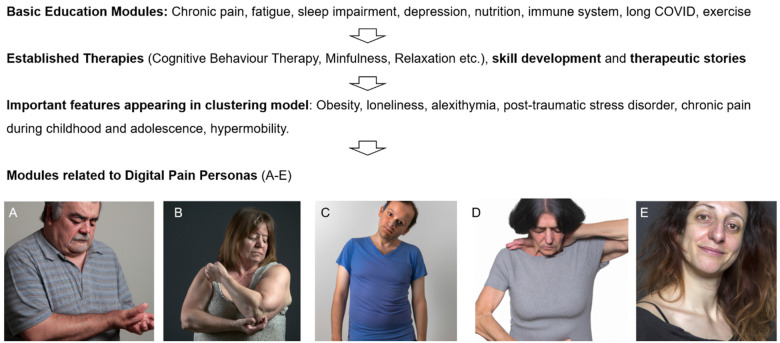
Flowchart of content creation. Initially, general therapy modules were developed on an interdisciplinary basis and recognized methods such as cognitive behavioral therapy were applied. Some of the modules of the online-program have been designed according results from unsupervised learning clusters and digital personas, as representative virtual profiles of chronic musculoskeletal pain phenotypes [adapted from (9)]. **(A)** Overweight male individual around 60 years with peripheral joint pain, typically nociceptive, often linked to conditions like osteoarthritis with low psychological comorbitidy or psychotropic medication. **(B)** Obese, often perimenopausal women in 45–55 years with severe sleep disturbances and low-grade inflammation. **(C)** Younger individuals with low BMI, alexithymia, and significant psychiatric issues. **(D)** Individuals on polymedicationafter complex medical histories including operations. **(E)** Women 20–40 with hypermobility, chronic pain (e.g., migraine) from childhood, and sometimes post-traumatic stress disorder.

The self-management program, developed collaboratively by rheumatologists, psychiatrists and physiotherapists, comprises 114 pages totaling 22,150 words, organized into 24 modules. It includes animated videos and physical exercise instruction videos. The program encompasses a variety of interventions, such as educational content on chronic pain, viral infections, long COVID, and the immune system, skill-building exercises, CBT, mindfulness practices, and therapeutic storytelling. Specific prevalent features identified in the five clusters, including alexithymia, sleep impairment, childhood pain, post-traumatic stress disorder (PTSD), migraine, obesity, and loneliness were targeted through dedicated modules. Additionally, several modules are tailored to each of the five digital “pain personas” representing specific combinations of symptoms, age, gender and psycho-somatic variables (Figure 1). An approximate word count for each intervention is provided in Table 1. It is important to note that this text-based analysis does not include the content of content of approximately 50 educational video files (physical exercise, relaxation, breathing instructions etc.), 53 figures, and 2 infographics.

**Table 1:** Characteristics of the mHealth self-management program.

Type of intervention	Word count (approx.)
Introduction and app usage instructions	500
Long Covid and post-viral fatigue basics	1,250
Fibromyalgia basics and general management	1,250
Depression	2,000
Genetic predisposition	200
Learning from other diseases	750
Chronic pain risk factors	2,000
Pain management Strategies	2,000
Understanding and supporting immune system	1,000
Stress management	1,500
Alexithymia	250
Nutritional insights and anti-inflammatory lifestyle	1,200
Mindfulness	1,000
Cognitive behavioral therapy	2,500
Hormonal factors	250
Sleep management	2,000
Therapeutic storytelling	2,000
Quizes	500

### Knowledge graph

For the knowledge graph analysis of our self-management program, we employed two tools: ChatGPT4 (OpenAI) and Infranodus (Nodus Labs, infranodus.com) as described elsewhere (11). The analysis began by embedding a PDF file of our entire program (except video files and images). For ChatGPT4, we utilized a straightforward prompt: “Perform a network analysis of this text”. Infranodus, on the other hand, offers a 3-dimensional text network visualization, where entities are depicted as nodes interconnected by edges. These edges can represent various types of relationships, such as causal, correlational, or hierarchical connections. The graphs are capable of illustrating complex semantic relationships, including abstract connections like conceptual similarities or historical ties. A comprehensive topical clustering analysis was conducted. This process highlighted the top relationships within the text, identifying them based on their significance and “betweenness” – a measure of how frequently they connect to other nodes in the network. The “occurrences” metric reflects the number of times a particular relationship appears within a 4-g (a sequence of four words) context. The “weight” of each relation indicates its relative importance within the text. “Betweeness” represents the degree to which nodes stand between each other (12).

### Gap-analysis and paraphrasing via ChatGPT4

A gap analysis was conducted using Infranodus as an integrated feature, focusing on the structure of the text network. This process involves comparing clusters of topics to identify missing connections, with the identified gaps being prominently highlighted in the knowledge graph. ChatGPT4 assists by suggesting terms that can bridge these knowledge gaps. These terms can then be expanded into full-text descriptions and seamlessly integrated into the knowledge graph to enhance its comprehensiveness.

### Content retro-engineering

We retro-engineered a knowledge graph for a similar self-management program in Infranodus without uploading the original text. We inserted specific prompts as described in the results. Using these prompts, a new knowledge graph was generated. Each node of this graph was then examined using the built-in ChatGPT4, which provided prompts for further exploration. A gap analysis was conducted on this newly created graph, and the knowledge graph was expanded as needed.

### Sentiment analysis

A total of 129 unique free text feedback entries (164 including repetitions; 9 UX feedback entries, 90 lines of “My Little Helper”, 65 lines of “Notes or comments”) were reviewed and categorized into thematic clusters UX feedback (feedback for improvement of the app and content), inputs from the “My Little Helper” app feature (what helped me), and notes or comments made by patients. Sentiment analysis mainly of French language was performed by Chat-GPT 4o to identify recurring themes and sentiments. The analysis involved creating a knowledge graph to visualize the relationships between key themes. The prompt aimed to build a global and easily readable representation of the main patient experiences, which was achieved by simplifying the graph for clarity. The final graph visually depicts connections between core themes, providing a high-level overview of the patient journey.

## Results

### Program development and digital personas

The development of the online program was conducted in four steps (Figure 1). First, modules were created to enhance understanding of chronic pain, fatigue, stress, sleep disturbances, the immune system, and Long COVID. This foundational information was essential for helping participants comprehend the complex interactions between these conditions. Second, the program incorporated techniques with known efficacy for fibromyalgia and chronic pain from other studies, particularly cognitive behavioral therapy, physical exercise, and relaxation. These methods were selected due to their established benefits in managing chronic conditions. To improve participant engagement and adoption of the techniques, various therapeutic stories were also integrated into the program, making the content more relatable and easier to apply. Third, based on the results of unsupervised learning clustering models, several key factors were prioritized in this online program (9). These factors include loneliness, obesity, alexithymia, depression, anxiety, PTSD, chronic pain during childhood and adolescence, perimenopause, sleep disturbances and hypermobility. Fourth, digital personas were created from the five identified clusters (Figures 1A–E). These personas were used to personalize the treatment approach. In the prediction model, Personas A–C were found to be most likely to respond positively to multimodal treatment and possibly to the digital treatment program. For example, Persona B is an overweight woman in her early 50s, experiencing joint and muscle pain (typically with bursitis) due to perimenopause, along with sleep disturbances and mild to moderate depression. This particular constellation of symptoms was addressed in various modules within the program, such as “Hormonal Changes”, “Nutritional Insights and Anti-inflammatory Lifestyle”, and “Obesity”. Cluster C represents a male persona around 30 years old, characterized by alexithymia and loneliness. This individual often has extensive screen time due to gaming or working in an IT profession and sometimes possesses high intellectual potential. The challenges associated with this persona were addressed in the modules “Alexithymia”, “Lonelyness” or “Gamification”. Persona D, on the other hand, is someone who receives polypharmacy, with multiple pain and psychotropic medications, such as high-dose opioids or trazodone, often combined with a history of multiple spinal surgeries. This phenotype was not prioritized in the program because it was associated with a very poor response to multimodal treatment. Persona E represents a relatively common phenotype in clinical practice, consisting of women aged 20–40 with hypermobility, back pain, migraines since childhood or adolescence, possibly PTSD, mild to moderate depression, and potentially endometriosis. The needs of this phenotype were addressed in various modules such as “Migraine”, “My Noisecard”, “PTSD”, and “Loneliness”.

### Knowledge graph by chatGPT

**Figure 2:**
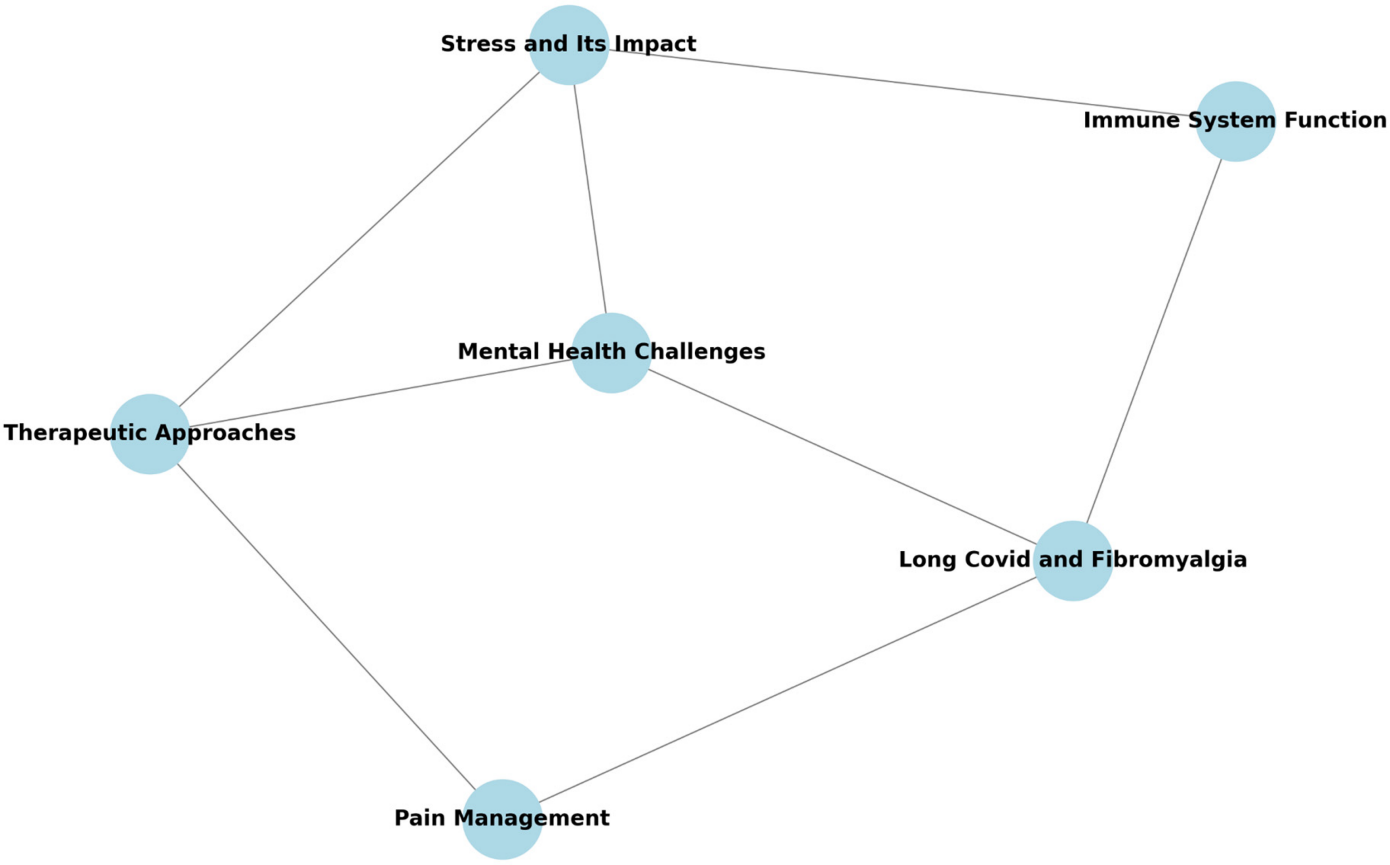
Simple knowledge graph illustration of the POCOS self-management program by ChatGPT.

The basic knowledge graph of POCOS, as revealed by ChatGPT-4, shows six interconnected nodes and their relationships (Figure 2). The identified topics include “**mental health challenges**” at the center, surrounded by “**stress and its impact**”, “**immune system function**”, “**long COVID and fibromyalgia**”, “**pain management**”, and “**therapeutic approaches**”.

### Graphical network analysis

**Figure 3:**
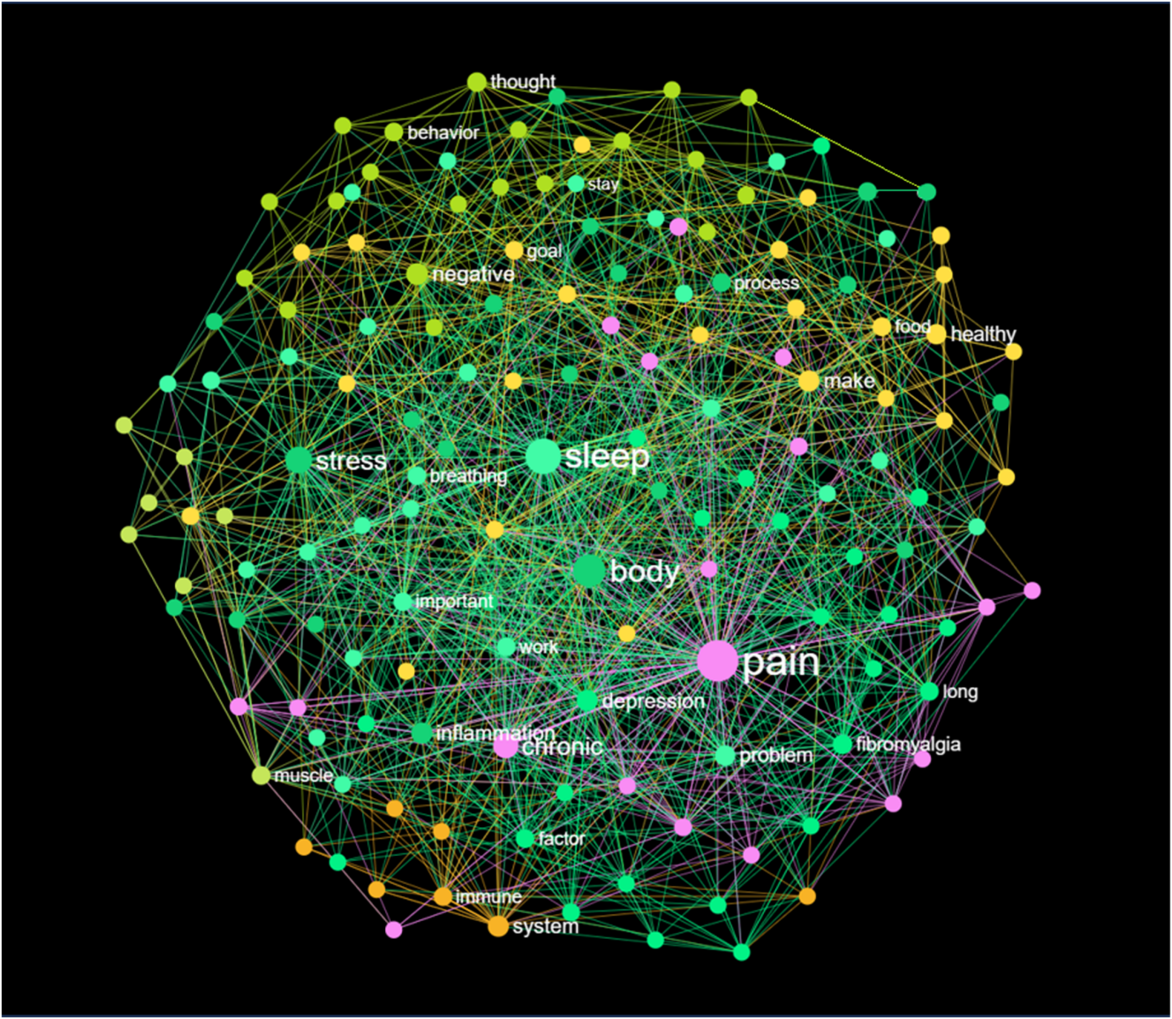
3D-knowledge graph of the self-management content by infanodus.

We utilized Infranodus to develop a more comprehensive knowledge graph of the self-management program, featuring 3D visualization (see Figure 3). The nodes “**Sleep**”, “**Body**”, and “**Pain**” are depicted as central and the most interconnected elements. The network related to pain, highlighted in pink, appears to encompass the central network, which consists of the terms “sleep”, “body”, and “stress”. The terms “chronic” and “pain” were the most closely connected in terms of weight, followed by “immune system” and “long COVID”. The highest betweenness centrality was observed between the pairs “pain” and “sleep”, “body” and “pain”, and “pain” and “stress” (Table 2).

**Table 2:** Top relations in 4-grams (most frequent co-occurrences of words in context) according to highest weight.

Source	Target	Occurrences	Weight	Betweenness
Chronic	Pain	68	326	0.6200
Immune	System	32	178	0.0935
Long	Covid	27	143	0.0282
Negative	Thought	18	122	0.1108
Back	Pain	22	116	0.4816
Inflammation	Body	18	108	0.3547
Body	Pain	41	101	0.7667
Stress	Negative	16	100	0.2468
Reduce	Inflammation	22	98	0.0717
Improve	Sleep	26	96	0.3487

### Main concepts and topical cluster analysis

The model pinpointed the primary concepts of the program based on four key terms: **pain, sleep, body, and stress.** In the knowledge graph, the highlighted concepts included “**body-mind**”, “**chronic pain**”, “**sleep-health**”, “**cognitive therapy**”, and “**mental health**”. A topical cluster analysis provided a more detailed overview of the self-management program (refer to Table 3). It revealed that the concept of “**chronic pain management**” had the greatest influence, followed by “sleep hygiene” and “immune system function”.

**Table 3:** Topical cluster analysis.

Topical cluster	Influence	Total nodes	Percentage of entries	Category	Keywords
1	26%	14	15%	1. Chronic pain management	Pain
2	19%	24	22%	2. Sleep hygiene	Sleep
3	18%	14	9%	3. Immune system function	Body
4	15%	19	15%	4. Cognitive therapy	Stress
5	9%	26	14%	5. Healthy eating	Healthy
6	7%	18	10%	6. Emotional development	Depression
7	4%	18	9%	7. Fibromyalgia causes	Fibromyalgia
8	1%	10	6%	8. Deep Relaxation	Muscle

### Gap analysis and paraphrasing

The model identified several conceptual gaps, e.g., between the terms “sleep” and “cognitive therapy”. To address this gap, ChatGPT4 paraphrased the following text:

ChatGPT: “If fatigue persists without another clear reason, consider these three pieces of advice: Regular exercise can improve your energy levels and muscle strength, reducing fatigue. However, it's crucial to listen to your body and avoid overexertion. Managing chronic stress is also important, as it can contribute to fatigue. Incorporate stress-reducing activities like meditation, deep breathing, or exercise into your routine. Remember to take things slow and not catastrophize. Communicate this to yourself and others, including your boss if necessary. Brain fog, or cognitive dysfunction, refers to a range of symptoms affecting cognitive function, such as difficulty concentrating, memory issues, and impaired decision-making”.

Other conceptual gaps identified were between “stress” and “negative behavior”, and “body”, “system”, and “inflammation”. A third blind spot was the connection between “healthy eating” and “positive thinking”. The model suggested developing content around terms like food, muscle, development, energy, and breathing. This is intriguing, as the program's authors focused on the anti-inflammatory aspects of food, but not on muscle development, which can be relevant in post-viral fibromyalgia.

### Analysis of selected concepts

**Figure 4:**
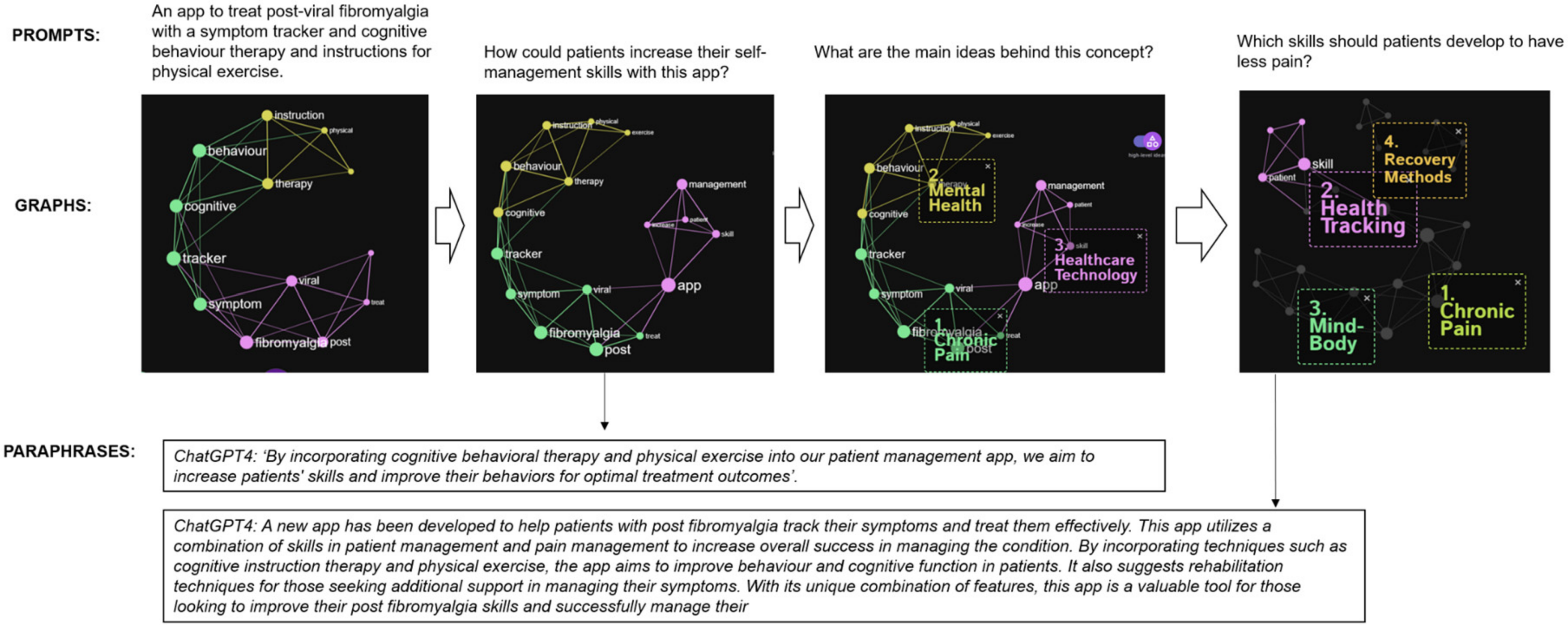
Prompt-based concept for a self-management content creation without uploaded PDF.

A selective concept relation analysis was conducted for various chosen nodes to elaborate on the synapses between concepts. For instance, within the knowledge graph, we examined the connection between “pain” and “covid”. This connection had a significance of 0.48 (accounting for 19% of the total influence) and was linked to 23 out of 150 nodes (representing 15% of the graph). ChatGPT suggested the following terms to bridge the gap between “pain” and “Covid”: “sleep”, “body”, “chronic”, “fibromyalgia”, “muscle”, and “breathing”. These suggestions are emphasized within the graph (see Figure 4). It's important to note that the breathing modules in this program, which were two animated videos, were not included in this analysis.

### Content retro-engineering

In this “retro-engineering” step, we constructed a new conceptual framework for a self-management program tailored for post-viral fibromyalgia-like syndrome. The model was provided with various prompts, but not the PDF of the existing program (Figure 4). We began with a general prompt: “An app to treat post-viral fibromyalgia with a symptom tracker, cognitive behavior therapy, and physical exercise instructions”. This led to the creation of 9 nodes, color-coded according to their connectivity and betweenness in the LLM. Following this, we prompted for ideas on skill development, which were subsequently integrated into the knowledge graph. Infranodus features a function titled “show main ideas behind this concept”. For our next prompt, we inquired about what patients can do to alleviate pain, which again accentuated the main concepts in the knowledge graph. The model recommended tracking/monitoring symptoms, managing chronic pain, learning recovery methods, and enhancing the mind-body axis through cognitive behavior therapy. At each stage of developing the knowledge graph, there is an option to generate paraphrases with ChatGPT-4.

### Sentiment analysis of patient response

**Figure 5:**
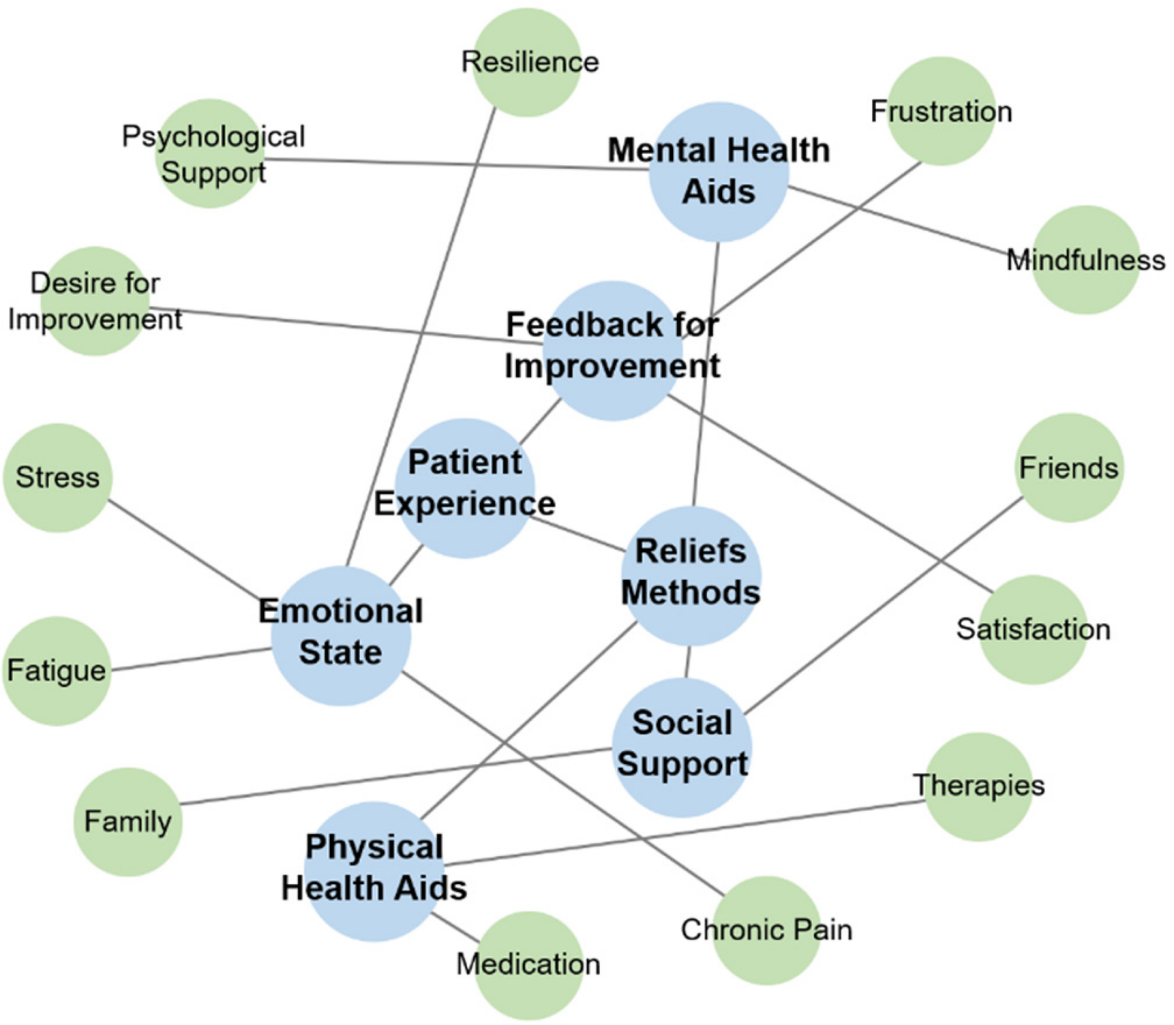
Knowledge graph of patient experience themes from outputs via the app and patient suggestions for improvements.

A total of 129 unique feedback entries (164 including repetitions; 9 UX feedback entries, 90 lines of “My Little Helper”, 65 lines of “Notes or comments”) were collected and used as input data for the sentiment analysis. The prompt aimed to create a knowledge graph providing a global and readable representation of the main patient experiences. Additional prompts were applied for further simplification of the graph. The final graph, shown in Figure 5, visually depicts the connections between core themes. Here the feedback was divided into propositions for app improvement and feedback on specific content, notably concerning psychological support and mindfulness, medication, and other therapies. Patients shared their emotional states and the effects of the app, primarily focusing on stress, fatigue, resilience, and chronic pain. Most of these topics align with the program content shown in Figure 3, though social support from family and friends was not addressed.

## Discussion

This study demonstrates that knowledge graphs can effectively summarize and conceptually analyze DTx programs. These automated semantic descriptions are valuable for characterizing online self-management programs, providing insights into clinical trial interventions and enhancing comparability across similar interventions. In other words, knowledge graphs serve as a powerful tool for increasing the transparency of DTx content, in a similar way to their use in molecular treatments (13).

The results show that our self-management program has a focus on pain, sleep, and stress management, revealing connections with other topics such as viral infection, inflammation, nutrition or depression. The program's origins in a rheumatology-led multimodal inpatient program for chronic musculoskeletal pain syndromes, primarily fibromyalgia, significantly influenced its content. The pandemic and its aftermath exacerbated symptoms in many of our fibromyalgia patients as reported in other cohorts (14). The program's content also drew from a survey conducted among long COVID patients to adapt to specific needs of those patients. As shown in the knowledge graph, however, the our program has a strong emphasis on fibromyalgia, more than on long COVID. Indeed, the program aligns with existing online fibromyalgia management programs that focus on CBT, pain coping skills, mindfulness, psychoeducation, physical exercise, lifestyle modification, and pain neuroscience education (5). However, the specific content or composition of each module in these programs remains unpublished. As such, knowledge graphs from other DTx programs could not be created.

As illustrated by the knowledge graph, our intervention had some characteristics, which was based on our data analysis and digital twin creation. As we found sleep impairment in a substantial part of our patients, we dedicated more content on this issue. We also found a considerable rate of peri- and post menopause, alexithymia (feeling blindness) and pain since childhood, we therefore dedicated a specific module for this. The creation of digital phenotypes proved helpful in developing persona-specific modules. For instance, one module explored the relationship between perimenopause and how the loss of anti-inflammatory estrogens can lead to musculoskeletal pain, increased weight, sleep impairment, and emotional imbalance, collectively resembling fibromyalgia. In fact we postulate that mHealth programs should generally take into account disease phenotypes, especially in heterogenic syndromes such as fibromyalgia (15).

One limitation of our study is the exclusion of video content and physical exercises from the analysis. In fact, voice transcription could capture this content for inclusion in future knowledge graphs. Additionally, we employed only two software tools, ChatGPT and Infranodus, for knowledge graph creation, despite the availability of numerous other options in medical data analysis (16). Prior research on DTx knowledge graphs is limited, with studies like InfraNodus mainly analyzing social media content (17). Furthermore, ChatGPT is not trained specifically on medical data, which represents another limitation.

In our work we also illustrate how knowledge graphs can enhance content through gap analyses. ChatGPT4 was instrumental in addressing these gaps and refining the program by focusing on the “synapses” between disease concepts. We found this function interesting, as those interconnections might be treatable by targeted self-management interventions, thus realing “drug-like” effects of the DTx. However, it's important to note that this AI-driven approach, while innovative, doesn't guarantee enhanced program efficiency, and confabulations may occur (18). Direct testing of AI-enabled or fully AI-created self-management interventions could be implemented in DTx and evaluated in decentralized clinical trials.

We combined knowledge graph software with ChatGPT to develop an online self-management program “from scratch”. The knowledge graph visualizations aided this “reengineering” process. A key limitation here is the nonspecific “ground truth” of the LLM model, which is not trained specifically on medical texts or self-management tools.

Finally, we compared knowledge graphs of the online program content (Figure 3) with a sentiment analysis of feedback from fibromyalgia patients who completed the online program (Figure 4). This feedback loop shows that most patient sentiments align with the content of the online program. However, social contacts in the form of friends and family were not addressed and will be included in the next version of the program. Patient outcomes will then be compared to those from the previous version.

The potential future role of multimodal AI in this context is significant, with newer applications enabling video and avatar creation through simple prompts. Before clinical efficacy and safety testing, user experience assessments of newly developed modules in form of an automated feedback loop could provide valuable insights, a frequently overlooked aspect in DTx. While it remains uncertain whether knowledge graphs can be of similar or more value for DTx than molecular therapies (19), they may uncover previously unknown connections between disease mechanisms, as demonstrated in our study on fibromyalgia. Integrating clinical data, as we have done, could further enhance this approach. In conclusion, AI-supported knowledge graphs could play a significant role in DTx at various levels, yet they cannot replace clinical trials or user experience research.

## Data Availability

The raw data supporting the conclusions of this article will be made available by the authors, without undue reservation.
